# Unraveling mucolipidosis type III gamma through whole genome sequencing in late-onset retinitis pigmentosa: a case report

**DOI:** 10.1186/s12886-023-03136-4

**Published:** 2023-09-26

**Authors:** Karl De Geer, Katarzyna Mascianica, Karin Naess, Eliane Sardh, Anna Lindstrand, Erik Björck

**Affiliations:** 1 Department of Molecular Medicine and Surgery, Karolinska Institutet, 17177 Stockholm, Sweden; 2 Department of Clinical Genetics, Karolinska University Hospital, 17177 Stockholm, Sweden; 3 Vitreoretinal Department, St. Erik Eye Hospital, Stockholm, Sweden; 4 Centre for Inherited Metabolic Diseases, Karolinska University Hospital, 17176 Stockholm, Sweden; 5 Department of Medical Biochemistry and Biophysics, Karolinska Institutet, 17177 Stockholm, Sweden

**Keywords:** Inherited retinal dystrophy, Retinitis pigmentosa, Whole genome sequencing, *GNPTG*, Mucolipidosis type III gamma, Lysosomal disease, Case report

## Abstract

**Background:**

We describe the case of a 47-year-old man referred to a retinal clinic and diagnosed with late-onset retinitis pigmentosa. Surprisingly, genetic testing revealed compound heterozygous pathogenic variants in *GNPTG*, leading to the diagnosis of the autosomal recessive lysosomal storage disorder mucolipidosis type III gamma. Mucolipidosis type III gamma is typically diagnosed during childhood due to symptoms relating to skeletal dysplasia. Retinal dystrophy is not a common phenotypic feature.

**Case presentation:**

Ophthalmologic examination was consistent with a mild form of retinitis pigmentosa and included fundus photography, measurement of best-corrected visual acuity, optical coherence tomography, electroretinogram and visual field testing. Extraocular findings included joint restriction and pains from an early age leading to bilateral hip replacement by age 30, aortic insufficiency, and hypertension. Genetic analysis was performed by whole genome sequencing filtered for a gene panel of 325 genes associated with retinal disease. Two compound heterozygous pathogenic variants were identified in *GNPTG*, c.347_349del and c.607dup. The diagnosis of mucolipidosis type III gamma was confirmed biochemically by measurement of increased activities of specific lysosomal enzymes in plasma.

**Conclusion:**

To our knowledge this is the first description of retinitis pigmentosa caused by compound heterozygous variants in *GNPTG*, providing further indications that late-onset retinal dystrophy is part of the phenotypic spectrum of mucolipidosis type III gamma.

## Background

Mucolipidosis type III gamma (MLIII gamma, MIM #252,605) is a rare, slowly progressive lysosomal storage disorder with autosomal recessive inheritance. The major phenotypic feature is skeletal dysplasia, and onset of clinical symptoms is seen during the first ten years of life. The most common presenting symptoms are joint stiffness (hips, shoulders, clawing of fingers), widening of the wrist joint, scoliosis and corneal clouding. Less common symptoms include short stature, coarse facial features, thickening of the skin, astigmatism, hyperopia, cardiac valve abnormalities, intellectual disability, organomegaly and cardiorespiratory problems [1–3]. Radiological findings include vertebral changes and dysostosis multiplex [3, 4]. Biochemically MLIII gamma is characterized by increased activities of lysosomal enzymes in plasma and reduced activities in cultured fibroblasts [5].

MLIII gamma is caused by biallelic pathogenic variants in the *GNPTG* gene (MIM #607,838) [6]. An overview of published cases from 2019 by Velho et al. identified 79 individuals with a molecular diagnosis of MLIII gamma. Among them 50 different disease-causing variants in *GNPTG* were observed [7]. The *GNPTG* gene encodes the gamma subunit of the enzyme *N*-acetylglucosamine-1-phosphotransferase (GlcNAc-1-phosphotransferase) which is involved in transport of newly synthesized hydrolases to the lysosome [8]. In MLIII gamma a subset of hydrolases is missorted and hypersecreted to the extracellular space, leading to accumulation of non-degraded macromolecules in dysfunctional and enlarged lysosomes [7].

Here we describe a case of late-onset retinitis pigmentosa where genetic testing surprisingly identified two pathogenic variants in *GNPTG*, leading to the diagnosis of MLIII gamma. Retinal dystrophy is an unusual finding in MLIII gamma and to our knowledge it has only been described once previously in a report from 2011 by Schrader et al. [9].

## Case presentation

A 47-year-old man of Swedish origin was referred to a retinal clinic with the suspicion of retinitis pigmentosa and epiretinal membrane (ERM) in both eyes. The patient reported decreased night vision and visual field narrowing starting from 45 years of age, but no metamorphopsias or visual impairment. His father had ERM. There was no other heredity for eye disease or skeletal diseases in first- or second-degree relatives.

**Fig. 1:**
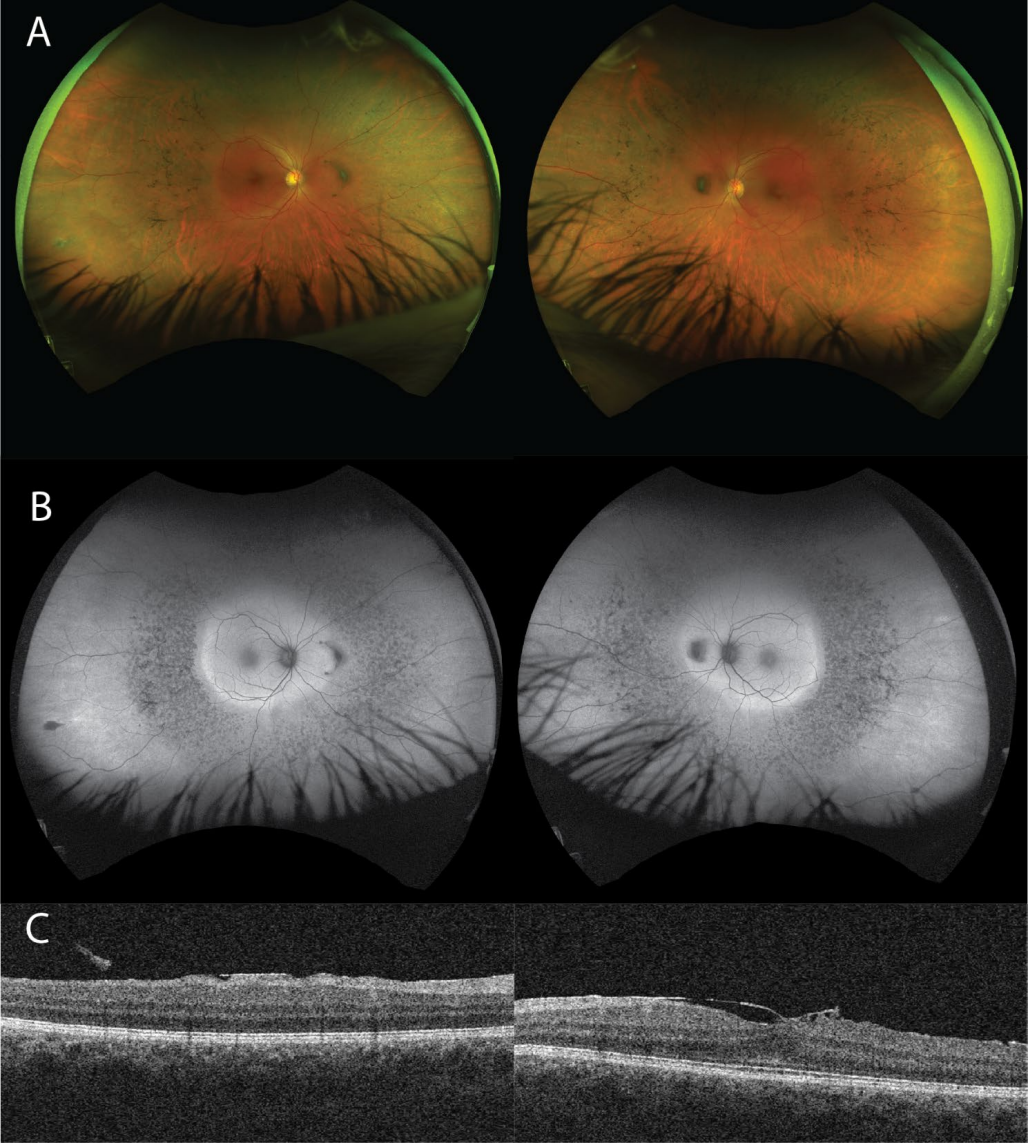
(**A**) Wide angle fundus photography, Optos. There is blunting of the foveal contour and scattered pigmentary changes in the middle periphery in the form of discrete bone-spicules and pigment clumping. (**B**) Wide angle fundus autofluorescence. The centre is well bounded. There is pathological patchy hypoautofluorescence in the middle periphery, and more homogenous hyperautofluorescence peripherally. (**C**) Optical coherence tomography. Extrafoveal scan at the right eye and foveal scan at the left eye. There is a mild epiretinal membrane with flattened foveal profile but without cystoid macular edema

**Fig. 2:**
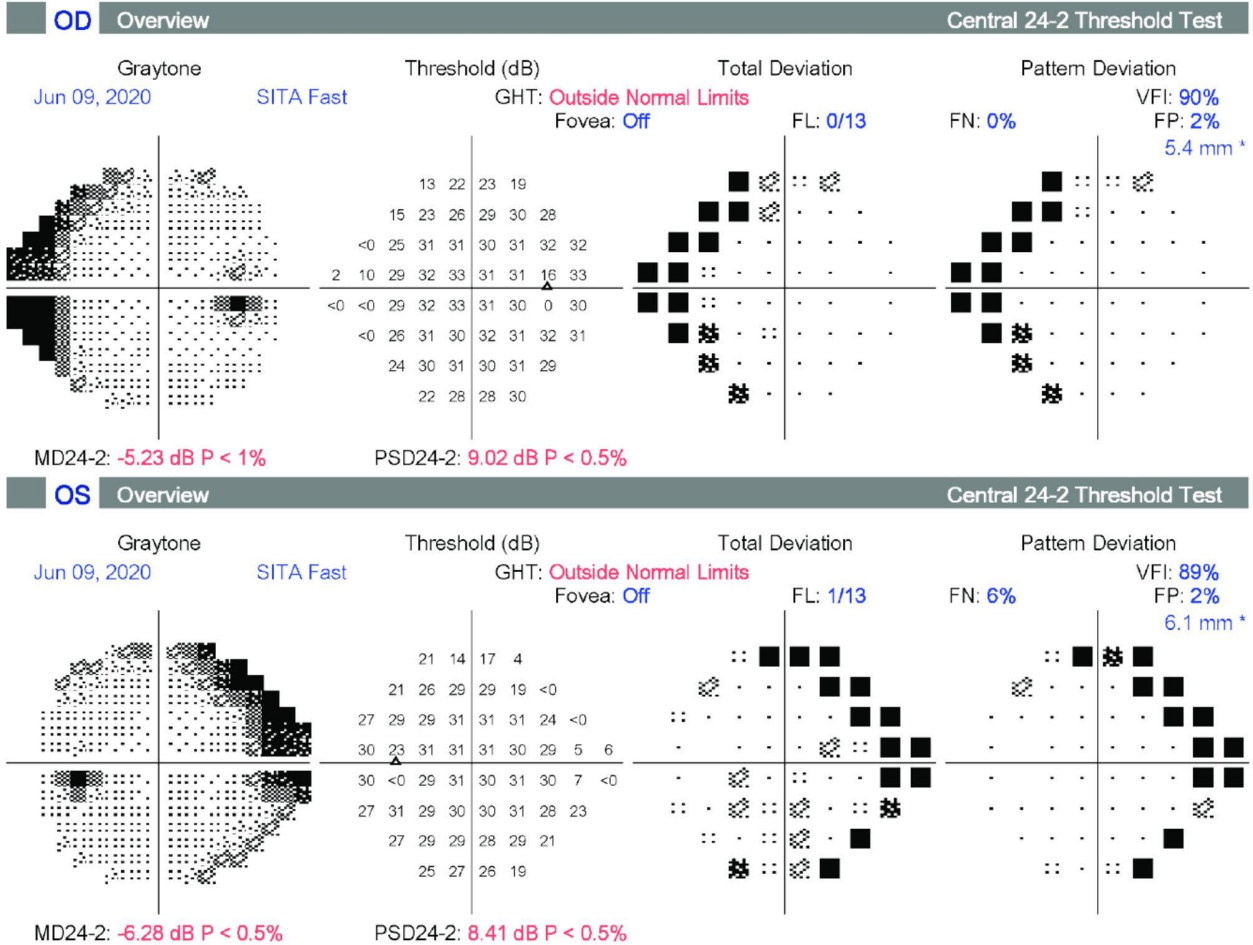
Threshold perimetry showing peripheral scotoma from the nasal site and well-preserved central sensibility

The best corrected visual acuity was 0.63 in both eyes (Snellen chart) and the Early Treatment Diabetic Retinopathy Study visual acuity was 78 and 75 for the right and the left eye. Examination of the anterior segment was normal with clear lens and no corneal opacity. The vitreous was clear except for a Weiss ring in both eyes. There was blunting of the foveal contour and scattered pigmentary changes were seen in the middle periphery in the form of discrete bone-spicules and pigment clumping (Fig. 1A). Fundus autofluorescence showed patchy hypoautofluorescence in the middle periphery and more homogenous hyperautofluorescence peripherally in both eyes (Fig. 1B). Macula cross section on optical coherence tomography showed light ERM with a slightly flattened foveal profile. Changes were more pronounced in the left eye, but without clinically significant cystoid macular edema (Fig. 1C). Full field electroretinogram on an Espion apparat with Dawson, Trick, and Litzkow electrodes showed moderate to significant amplitude reduction with both scotopic and photopic stimulation. The rod-system was more affected than the cones. Threshold perimetry 24:2 showed peripheric restriction nasally up to 20 degrees and Goldmann kinetic perimetry, which was performed later, confirmed the peripheral narrowing of the visual field (Fig. 2). A clinical diagnosis of a mild form of retinitis pigmentosa was made and the patient was referred for genetic testing.

Whole genome sequencing was performed and analyzed as previously described [10, 11]. Data was analyzed using a panel of 325 genes associated with retinal disease. Two variants previously reported as pathogenic were identified in *GNPTG*, c.347_349del and c.607dup (Table 1). Segregation analysis confirmed the presence of one variant in each parent, consistent with autosomal recessive inheritance. The finding was unexpected, since retinitis pigmentosa is not commonly reported as part of the phenotypic spectrum of MLIII gamma.

**Table 1:** Whole genome sequencing revealed two heterozygous variants previously reported as pathogenic

Variant	Consequence	Previous reports
c.347_349del (p.Asn116del)	In-frame deletion	Tiede et al., 2004, Persichetti et al., 2009, Tuysuz et al., 2018
c.607dup (p.Gln203fs)	Loss-of-function	Raas-Rothschild et al., 2004

However, scrutiny of the medical history revealed additional findings compatible with MLIII gamma (Table 2). The patient had suffered from joint pains and gait abnormality from two years of age. Radiological images were not available but medical records note a hypoplastic L1 vertebra and pronounced lordosis at age 20. The patient underwent bilateral hip replacement surgery at age 30 and bilateral osteoarthritis surgery of several metatarsophalangeal joints at age 40. A mild nonprogressive aortic insufficiency was discovered at age 23. Echocardiography at age 43 showed a structurally normal heart with a tricuspid aortic valve. The left ventricle had normal dimensions and normal systolic function with an estimated ejection fraction of 60%. At age 35 years the patient was diagnosed with hypertension, treated with an ACE-inhibitor. The patient had no intellectual disability. Physical measurements included a weight of 50.4 kg and height of 169.5 cm. The arm span was 184 cm and the symphysis pubis to floor distance was 89.5 cm. The upper/lower segment ratio was 0.89 (reference value for reduced ratio < 0.85) and the arm span/height ratio was 1.09 (reference value for increased ratio > 1.05) [12].

**Table 2:** Clinical characteristics of the patient. Common findings in MLIII gamma not observed in the patient included coarse face, corneal clouding, hand deformities, hardening of skin, scoliosis, and short stature

Clinical characteristics	Onset, years
**Common findings**	
Gait abnormality and joint pains	2
Aortic insufficiency	23
Bilateral hip replacement	30
Surgery of metatarsophalangeal joints	40
**Rare findings**	
Hypertension	35
Epiretinal membrane	47
Retinitis pigmentosa	47

Biochemical analyses to confirm the diagnosis were performed. The activities of the lysosomal enzymes hexosaminidase A and B, alfa-fucosidase, beta-galactosidase and beta-glucuronidase in plasma and leukocytes were determined by methods using the hydrolysis of synthetic substrates based on 4-methylumbelliferyl. The amount of 4-methylumbelliferon (4-MU) was measured using fluorometry [13–16].

The activities of certain lysosomal enzymes were highly increased in plasma, compared to the average activity of three healthy age matched controls (Table 3). Activities in leukocytes for the same enzymes were slightly decreased or within lower normal range when compared to locally established reference intervals based on 20 healthy adults (data not shown). The biochemical pattern is consistent with a mucolipidosis.

**Table 3:** Biochemical analyses revealed increased activities of certain lysosomal enzymes in plasma of the patient, compared to the average of three age-matched healthy controls. µkat/L = microkatals per liter

Lysosomal enzyme	Patient (µkat/L)	Control (µkat/L)	Ratio
α-fucosidase	170	160	1.1
β-galactosidase	4.2	0.9	4.7
β-glucuronidase	1370	42	33
Hexosaminidase A	1400	280	5.1
Hexosaminidase B	5190	135	38

Although the patient’s subjective symptomatology remained stable one year after clinical diagnosis, the ophthalmological examination indicated a progressive deterioration of the visual field; threshold perimetry showed a reduction in the Visual Field Index, 90–70% (right eye) and 89–69% (left eye), whereas fundus autofluorescence and optical coherence tomography remained stable. The patient is followed-up by a multidisciplinary team and has received genetic counseling. The patient has given informed consent for publication.

## Discussion and conclusions

To our knowledge this is the first description of retinal disease caused by compound heterozygous variants in the *GNPTG* gene. Both variants identified in our patient have been previously described in patients with MLIII gamma, but only in homozygous form and never in combination with retinal disease. The variant c.347_349del (p.Asn116del) leads to an in-frame deletion of asparagine at position 116, which constitutes one of two potential *N*-glycosylation consensus sequences in the protein. It has been associated with a milder phenotype and has been reported in six homozygous patients [17–19]. The variant c.607dup (p.Gln203fs) leads to a premature stop codon and has been described in four homozygous patients of a Turkish consanguine family. Variable expressivity was observed, ranging from the classical phenotype of joint restriction to isolated hip involvement and two asymptomatic individuals [1].

Recent reviews and larger cohorts make no mention of retinal disease as part of the phenotypic spectrum of MLIII gamma [3, 4]. *GNPTG*-related retinitis pigmentosa has to our knowledge only been described once previously, in a large consanguine Canadian family carrying the homozygous variant c.238_243del (p.Lys80_Tyr81del). Seven of the ten affected individuals presented in the third and fourth decade with decreased night vision, which progressed to significant peripheral and central vision loss or blindness by the fifth to seventh decade [9]. Other retinal abnormalities have been described in mucolipidosis type III patients, though most such descriptions are from older publications before genetic testing could differentiate MLIII gamma from the related, more severe disease mucolipidosis type III alpha/beta (MLIII a/b). Traboulsi & Maumenee described four patients ranging from 4 to 18 years old, two with moderate venous tortuosity and epiretinal membrane formation, and two with mild retinal haze and a milky appearance of the retina. One of the patients had a superior sector of bone spiculae formation in one eye with a corresponding visual field defect. They concluded that mild and very slowly progressive retinopathy seemed to be within the phenotypic spectrum of MLIII [20]. Pourjavan et al. described one patient with mild retinal vascular tortuosity and mild epiretinal membrane formation at age 13 [21] Of note, we observed similar mild structural changes of the macula in our patient. The changes were not associated with visual symptoms and did not require treatment.

The median age at diagnosis of MLIII gamma is nine years [4]. Our patient as well as those described by Schrader et al. developed symptoms of retinitis pigmentosa between the third and fifth decade, which indicates that late-onset retinal dystrophy could be an overlooked feature of MLIII gamma. This is supported by findings in mouse models. MLIII gamma is caused by dysfunction of the gamma subunit of the enzyme GlcNAc-1-phosphotransferase. Mice with absent GlcNAc-1-phosphotransferase activity are born with normal retinas but develop a relatively late-onset progressive retinal degeneration characterized by loss of inner and outer segments of the photoreceptors and of the outer nuclear layer, leading to blindness by three months of age [22, 23]. In humans absent activity of GlcNAc-1-phosphotransferase cause mucolipidosis type II (MLII), a severe disease which is usually lethal during childhood, and not known to be associated to retinal disease [4]. The relatively normal life span of mice compared to humans indicate that retinal disease could be an age-related phenotypic trait and might explain why retinal disease has only been observed in MLIII gamma, and not in the related but more severe diseases MLII and MLIII a/b.

In conclusion, we describe the first case of retinitis pigmentosa caused by compound heterozygous variants in *GNPTG*, providing further indications that late-onset retinal dystrophy is part of the phenotypic spectrum of MLIII gamma. Since MLIII gamma is usually diagnosed during childhood, it is important for the clinician to be aware of possible development of retinal disease later in life.

## Data Availability

The dataset used/analyzed in the current study is not publicly available due to the European GDPR law but are available from the corresponding author on reasonable request. The genetic variants reported in this case report will be submitted to the ClinVar Database, to be accessed by the following links: NM_032520.5(GNPTG):c.607dup (p.Gln203fs), accession VCV000553164.9: https://www.ncbi.nlm.nih.gov/clinvar/variation/VCV000553164.9 (accessed Feb. 2, 2023). NM_032520.5(GNPTG):c.344ACA[1] (p.Asn116del), accession VCV000021715.22: https://www.ncbi.nlm.nih.gov/clinvar/variation/VCV000021715.22 (accessed Feb. 2, 2023).

## Abbreviations

MLIII: Gamma Mucolipidosis type III gamma
ERM: Epiretinal membrane
µkat/L: Microkatals per litre
MLIII: a/b Mucolipidosis type III alpha/beta
MLII: Mucolipidosis type II
